# Less Is More – Estimation of the Number of Strides Required to Assess Gait Variability in Spatially Confined Settings

**DOI:** 10.3389/fnagi.2018.00435

**Published:** 2019-01-21

**Authors:** Daniel Kroneberg, Morad Elshehabi, Anne-Christiane Meyer, Karen Otte, Sarah Doss, Friedemann Paul, Susanne Nussbaum, Daniela Berg, Andrea A. Kühn, Walter Maetzler, Tanja Schmitz-Hübsch

**Affiliations:** ^1^Charité – Universitätsmedizin Berlin, Corporate Member of Freie Universität Berlin, Humboldt-Universität zu Berlin, and Berlin Institute of Health, Department of Neurology, Berlin, Germany; ^2^Department of Neurology, Universitätsklinikum Schleswig-Holstein, Kiel, Germany; ^3^Department of Neurodegenerative Diseases, Center for Neurology, Hertie Institute for Clinical Brain Research, Tübingen, Germany; ^4^Charité – Universitätsmedizin Berlin, Corporate Member of Freie Universität Berlin, Humboldt-Universität zu Berlin, and Berlin Institute of Health, Neurocure Cluster of Excellence, Berlin, Germany; ^5^Experimental and Clinical Research Center, Max Delbrück Center for Molecular Medicine and Charité – Universitätsmedizin Berlin, Berlin, Germany; ^6^Berlin School of Mind and Brain, Charité – Universitätsmedizin Berlin, Berlin, Germany

**Keywords:** gait variability, gait analysis, turn detection, healthy elderly, movement disorders

## Abstract

**Background:** Gait variability is an established marker of gait function that can be assessed using sensor-based approaches. In clinical settings, spatial constraints and patient condition impede the execution of longer distance walks for the recording of gait parameters. Turning paradigms are often used to overcome these constraints and commercial gait analysis systems algorithmically exclude turns for gait parameters calculations. We investigated the effect of turns in sensor-based assessment of gait variability.

**Methods:** Continuous recordings from 31 patients with movement disorders (ataxia, essential tremor and Parkinson’s disease) and 162 healthy elderly (HE) performing level walks including 180° turns were obtained using an inertial sensor system. Accuracy of the manufacturer’s algorithm of turn-detection was verified by plotting stride time series. Strides before and after turn events were extracted and compared to respective average of all strides. Coefficient of variation (CoV) of stride length and stride time was calculated for entire set of strides, segments between turns and as cumulative values. Their variance and congruency was used to estimate the number of strides required to reliably assess the magnitude of stride variability.

**Results:** Non-detection of turns in 5.8% of HE lead to falsely increased CoV for these individuals. Even after exclusion of these, strides before/after turns tended to be spatially shorter and temporally longer in all groups, contributing to an increase of CoV at group level and widening of confidence margins with increasing numbers of strides. This could be attenuated by a more generous turn excision as an alternative approach. Correlation analyses revealed excellent consistency for CoVs after at most 20 strides in all groups. Respective stride counts were even lower in patients using a more generous turn excision.

**Conclusion:** Including turns to increase continuous walking distance in spatially confined settings does not necessarily improve the validity and reliability of gait variability measures. Specifically with gait pathology, perturbations of stride characteristics before/after algorithmically excised turns were observed that may increase gait variability with this paradigm. We conclude that shorter distance walks of around 15 strides suffice for reliable and valid recordings of gait variability in the groups studied here.

## Introduction

Impairment of gait function is frequent in neurological disorders and in the aging population (Snijders et al., 2007; Jahn et al., 2015; Masdeu, 2016; Schlenstedt and Maetzler, 2016). It is associated with impairment of everyday mobility, increased risk of falling and thus impacting individuals’ quality of life. Gait disorders pose a challenge for clinical evaluation. Instrumented gait analysis offers the opportunity to quantify an abundance of parameters to describe and differentiate gait disorders (Pradhan et al., 2015). The use of wearable inertial measurement units may improve the clinical applicability of gait analysis and enable the collection of a large number of strides during continuous walking outside of a gait lab. A potential drawback of such systems is that their clinical application has to rely on inherent algorithms of gait segmentation that are usually not disclosed to the user. We therefore explored the potential to reliably measure an important gait feature – magnitude of step-to-step variability – using a commercially available sensor-based gait analysis system in the clinical setting. Previous data suggest validity of the gait analysis system we used in the populations of our study (Mancini et al., 2011; Horak and Mancini, 2013; Schmitz-Hubsch et al., 2016).

Gait variability is increasingly recognized as a diagnostically useful and clinically meaningful parameter. Irregular gait patterns have long been described as clinical features of specific disorders, such as cerebellar ataxia (Dichgans, 1984), but only quantitative assessment has objectively shown irregularity of stepping in a variety of conditions (Moon et al., 2016). The physiological variability observed in forward stepping, i.e., stride-to-stride fluctuations of scaling and timing during steady-state walking here throughout referred to as gait variability, has been interpreted as an indirect expression of dynamic motor control within the specific biomechanical constrains of human walking (Collins and Kuo, 2013; Wuehr et al., 2014a). Besides this adaptive component, which can be more directly expressed by stability measures (Hamacher et al., 2011), gait variability is considered to also contain portions of neuromuscular noise known to increase, e.g., with ageing (Bruijn et al., 2013; Roos and Dingwell, 2013). An increased magnitude of such variability may be due to disturbance on different levels, e.g., disturbed “internal clock” in basal ganglia disorders (Rao et al., 2014; Avanzino et al., 2016), as a consequence of disturbed coordination of limb muscle activity as e.g., with spasticity (Kao et al., 2014) or secondary to impaired balance in cerebellar disease (Morton and Bastian, 2003). Moreover, lower boundaries of gait variability have been delineated for normal walking (Gouelle et al., 2013; Konig et al., 2016b) but the interpretation of such findings is less clear (Beauchet et al., 2009) and we are aware of only very few reports (Brach et al., 2005; Rennie et al., 2017) that describe reduced gait variability as a possibly useful risk marker. In this paper we therefore focus on practical issues when screening for increased gait variability, considering different disease entities.

Different metrics have been used to describe fluctuations of forward stepping movements (Hamacher et al., 2011; Bruijn et al., 2013; Riva et al., 2014). Recent reviews defined stride time variability, which expresses the magnitude of variability as the coefficient of variation (CoV), as the most prevalent measure of gait variability among clinical studies (Konig et al., 2016a,b; Moon et al., 2016). Meta-analyses including more than 1000 (healthy and diseased) subjects showed consistent findings among studies and a value of 2.6% [2.3–3.1] has been proposed as a reliable upper limit for CoV stride time in physiological gait (Konig et al., 2016b).

Increased CoV of stride time, often accompanied by slowed walking speed, has been associated with decreased mobility, increased risk of falling, fear of falling, feeling of unsteadiness in different conditions (Schaafsma et al., 2003; Konig et al., 2014b; Wuehr et al., 2014b; Moon et al., 2015; Kalron, 2016; Lord et al., 2016) and with freezing of gait in Parkinson’s disease (PD) (Hausdorff et al., 2003). Recent studies established relations of this measure with CNS structural changes (Rosso et al., 2014; Tian et al., 2017; Corporaal et al., 2018). Increased gait variability has been described in pre-manifesting/early stages of different neurological conditions such as hereditary ataxias (Rochester et al., 2014; Ilg et al., 2016), familial Parkinsonism (Mirelman et al., 2013) and multiple sclerosis (Sosnoff et al., 2012). This motor feature occurred even in the absence of reduced gait speed or other clinical findings. This supports using the magnitude of gait variability as a screening measure for incipient neurological conditions in their prodromal stages. The clinical relevance is further supported by use of gait variability as the primary outcome in recent interventional trials (Beauchet et al., 2014; Henderson et al., 2016). In contrast, data on its biometric properties are scarce (Lord et al., 2011b).

Its repeatability was mostly reported for within-session retest (Brach et al., 2008; Paterson et al., 2009; Faude et al., 2012; Galna et al., 2013; Wittwer et al., 2013; Konig et al., 2014a; Schmitz-Hubsch et al., 2016) with only few reports on inter-session reliability (Faude et al., 2012; Galna et al., 2013; Wittwer et al., 2013; Konig et al., 2014a). It is obvious that single stride perturbations have larger effects on stride CoVs than on averages of spatiotemporal parameters themselves. In line with this, the reliability of gait variability measures was found much lower than the notably excellent repeatability of spatiotemporal gait parameters such as walking speed and this holds true for different re-test intervals and for healthy as well as diseased populations. Thus, recording more than 50 gait cycles considering only steady-state walking is commonly recommended (Konig et al., 2014a) for a reliable description of gait variability.

However, two aspects limit the collection of gait data over the proposed distance in realistic clinical settings: First, time constraints within the clinical setting prohibit transfer to a gait lab and spatial constraints on the ward most often allow only serial short-distance walks back and forth potentially increasing gait variability due to difference between successive walking bouts. Second, subjects with neurological conditions might get fatigued or feel unable to complete longer walking distances, which may confound the performance of the task, especially if test repetitions are required.

We therefore sought to explore how the magnitude of gait variability can be reliably captured in the clinical setting using a commercially available sensor-based gait analysis system. We specifically explored effects of the common practice to collect strides over several walking segments separated by 180° turns using an automated turn detection. To account for possible differences in the applicability and limitations of this testing paradigm with gait pathology, we included data from a large cohort of elderly healthy (HE) subjects and data from subjects with different movement disorders (MD) known to be associated with increased gait variability, namely cerebellar ataxia (ATX), essential tremor (ET) and Parkinson’s disease (PD).

## Methods, Subjects and Clinical Assessments

### Study Populations

Analyses were performed on two datasets of subjects with expected differences in gait variability.

The first dataset (MD dataset) comprised 31 subjects with movement disorders associated with increased gait variability [12 Parkinson’s disease (PD, age 60 ± 9), 7 cerebellar ataxia (ATX, age 58 ± 7), 12 essential tremor (ET, age 67 ± 10)] These subjects underwent gait analysis at the movement disorders clinic of Charité - Universitätsmedizin Berlin. Clinical details are provided in Table 1. Patients requiring walking aids or suffering from concurrent conditions with potential affection of gait (i.e., neuropathy, musculoskeletal impairments, vestibular disorders) were excluded. The study protocols were approved by the IRB of Charité - Universitätsmedizin Berlin (EA1/267/12, EA2/016/16, EA2/015/16, EA2/186/16).

**Table 1:** Clinical characteristics of patients with movement disorders.

Patient	Sex	Diagnosis	Age [years]	Disease Duration [years]	Rating instrument	Clinical score	Weight [kg]	Height [m]	Condition specific medication (daily dose)
ET01	M	ET	57	44	TRS	7/116	80	1.78	600 mg gabapentine 25 mg amitryptiline
ET02	F	ET	72	57	TRS	15/116	75	1.65	47.5 mg metoprolol
ET03	M	ET	63	50	TRS	26/116	84	1.78	100 mg propranolol 250 mg primidone
ET04	F	ET	71	30	TRS	10/116	68	1.72	None 100 mg pregabaline
ET05	M	ET	73	10	TRS	7/116	64	1.63	None
ET06	M	ET	53	39	TRS	30/116	72	1.73	None
ET07	F	ET	70	30	TRS	13/116	69	1.67	None
ET08	F	ET	77	8	TRS	12/116	63	1.52	None
ET09	F	ET	72	38	TRS	14/116	65	1.67	160 mg propanolol
ET10	M	ET	82	16	TRS	20/116	76	1.76	None
ET11	M	ET	48	18	TRS	38/116	75	1.87	250 mg primidone 50 mg propanolol
ET12	F	ET	70	12	TRS	32/116	63	1.63	120 mg propanolol
PD01	M	PD	64	5	UPDRS-III	16/108	90	1.84	n.a.
PD02	M	PD	73	21	UPDRS-III	12/108	86	1.85	2.1 mg pramipexol 150 mg levodopa + benserazide 100 mg amantadine
PD03	M	PD	58	21	UPDRS-III	20/108	91	1.88	600 mg levodopa + benserazide 300 mg amantadine
PD04	M	PD	56	11	UPDRS-III	18/108	85	1.8	None
PD05	M	PD	68	16	UPDRS-III	19/108	87	1.84	400 mg levodopa + benserazide 16 mg rotigotine
PD06	M	PD	57	20	UPDRS-III	12/108	69	1.73	800 mg levodopa + benserazide 50 mg safinamide
PD07	F	PD	62	n.a.	UPDRS-III	n.a.	n.a.	1.78	n.a.
PD08	M	PD	46	11	UPDRS-III	18/108	65	1.52	1.3 mg pramipexole 600 mg levodopa + benserazide 200 mg amantadine
PD09	F	PD	44	2	UPDRS-III	10/108	58	1.63	1 mg rasagiline 2 mg ropinirole
PD10	M	PD	67	8	UPDRS-III	19/108	67	1.68	850 mg levodopa + carbidopa 1000 mg entacapone 50 mg safinamid
PD11	F	PD	51	4	UPDRS-III	11/108	69	1.67	200 mg levodopa + benserazide 4 mg rotigotine 1 mg rasagiline
PD12	M	PD	69	4	UPDRS-III	17/108	70	1.72	600 mg levodopa + benserazide 6 mg rotigotine
ATX01	M	SCA14	64	21	SARA	8.5/40	86	1.78	None
ATX02	M	SCA14	60	13	SARA	12/40	93	1.75	None
ATX03	F	Cerebellar Ataxia	57	3	SARA	3.5/40	64	1.66	None
ATX04	F	Cerebellar Ataxia	66	6	SARA	6/40	56	1.53	None
ATX05	M	Cerebellar Ataxia	47	18	SARA	15/40	68	1.76	None
ATX06	F	Cerebellar Ataxia	61	5	SARA	8/40	65	1.58	None
ATX07	F	Cerebellar Ataxia	52	2.5	SARA	16/40	67	1.68	None

*ET – essential tremor; PD – Parkinson’s disease; MD – patients with movement disorders; SARA – Scale for Assessment and Rating of Ataxia; SCA14 – spinocerebellar ataxia genotype 14; TRS – Fahn-Tolosa-Marin Tremor Rating Scale (items 1–14); UPDRSIII – Unified Parkinson’s Disease Rating Scale part III (motor part).*

The second dataset (HE dataset) consisted of 172 healthy elderly individuals (78 females, average age 70.1 years ± 6.2) assessed during the third visit (2013/14) of the TREND study (Salkovic et al., 2017). Only subjects without functionally relevant disturbance of balance or locomotor function were included. The TREND study was approved by the ethics committee of the Medical Faculty of the University of Tübingen (Nr. 90/2009BO2). All subjects of both cohorts provided informed consent.

### Assessments

#### Clinical Assessment

The motor part of the Unified Parkinson’s Disease Rating Scale (UPDRS-III) (Jankovic and Tolosa, 2007) was used for the assessment of disease severity in PD, the Fahn-Tolosa-Marin Tremor Rating Scale items 1-14 (TRS) (Fahn et al., 1988; Stacy et al., 2007) for ET, and the scale for the assessment and rating of ataxia (SARA) (Schmitz-Hubsch et al., 2006) for cerebellar ataxia.

#### Gait Assessments

Gait was recorded in both studies (MD and HE) with a commercially available gait analysis system (Mobility Lab^®^, APDM, Portland, OR, United States) consisting of six body-worn inertial sensors, symmetrically attached to wrists, shanks and medially placed over sternum and lower back. In all patient groups, participants walked a 10-m distance ( = segment) five times back and forth at their preferred speed without specific instructions for turning. Two lines of colored tape orthogonal to the walking direction indicated the boundaries of the segment and provided a visual clue for turning. The dataset thus includes 50 m of walking and four turns of 180°. In the HE study, participants walked a 20-m distance (=segment) back and forth for 1 min, also at preferred speed and without specific instructions for turning but with respective segment ends marked with pylons.

### Data Processing and Statistical Analysis

At least 40 gait cycles were obtained per participant and included for analyses to ensure comparability across the groups and datasets.

In the first approach, we used the algorithms for turn excision provided by the manufacturer (Mobility Lab software V1.0.0.201503302135) to export raw data. Software output settings were preset to exclude turns from analysis. This yields export of stridewise timecoded values of all gait parameters from all segments of walking in between turns as defined by manufacturer’s turn excision. Of these, stride length and stride time (=gait cycle time) and their CoVs [(SD/mean)^∗^100] were used for further analysis.

**FIGURE 1:**
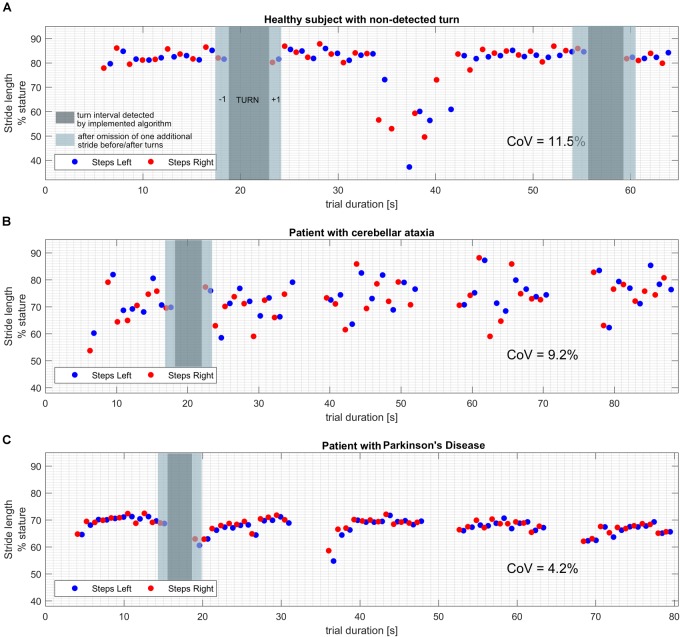
Exemplary plots of stride length values over the time course of the trial. Turns can be identified as gaps (dark gray overlay) in the timeseries as they are segmented and excluded by the implemented algorithm. Additional strides that were removed around turns in the alternative turn segmentation approach are marked with a lighter gray overlay. **(A)** Healthy subject with a turn that was not segmented by the software algorithm. Note the drastically shorter steps while turning, resulting in CoV beyond physiological range when calculated from all strides. **(B)** Patient with cerebellar ataxia and proper detection of all four performed turns. Note the pronounced fluctuation of stride length values from stride to stride, resulting in a CoV beyond physiological range. **(C)** Patient with idiopathic Parkinson’s disease and proper detection of all four performed turns. Note the decreased average stride length and the shorter strides right after the turns which may correspond to impaired step initiation.

For each individual trial, lengths and times of the strides were plotted against their respective time stamps (Figures 1A–C). This allowed us (1) to detect turns as “gaps” in the time-series, (2) to identify strides that occurred directly before and after such a turn and (3) to exclude trials with non-detection of turns that would show as irregular patterns of gaps or absence of such gaps in time series with corresponding implausible aberrations of stride length and time values.

To further evaluate the effect of algorithmic turn excision, we extracted gait parameters for strides directly before and after turns as defined by manufacturer’s algorithm. These strides were normalized to individual mean, expressed as percentage of mean of all 40 strides. In HE datasets, only the first two turns were used as this was the minimum performed by every participant. Histograms for strides before and after turns were produced for relative stride values and normalized for their probability distribution to account for different sample sizes. This renders frequency of occurrence as proportion of total counts per group. From group means and standard deviations, the probability density function for normal distribution was produced for each parameter and superimposed on the histogram to visualize skews. MD were treated as one group for this visualization while parameter values for stride length and time before and after turn were additionally calculated by disorder (Supplementary Table S4).

Comparisons between characteristics of stride before and after turns and averages from total distance were conducted with paired t-tests. Results were not compared between groups due to the large differences in sample size.

As a second approach – as results suggested a relevant difference in stride characteristics before/after turns versus average of all strides in a substantial proportion of subjects (Supplementary Table S3) –we used a more generous turn segmentation that excluded one additional stride before and after each automatically segmented turn. All further analysis was replicated using this alternative approach to explore if a more “generous” turn segmentation would attenuate any confounding effect of turns on stride length/time CoVs. This approach resulted in a lesser total number of strides and thus analyses were referenced to a maximum number of 32 strides in MD and HE.

In order to explore the dependency of CoVs from the number of strides recorded, we calculated individual CoVs for (a) each of the five/three (MD/HE) segments of straight 10/20 m walking between turns and (b) cumulative for each of 3–40 strides when using manufacturer’s turn excision and 3–32 when applying the alternative turn segmentation.

Results of (a) were used to determine the intra-individual consistency of spatial and temporal gait parameters and their CoV. A two-way random single measure model was applied to compute intraclass coefficient (ICC) with segments regarded as “repeated measurements.” Thus ICCs reflect to which degree individuals maintain their results stable over the 3–5 segments of continuous walking segmented by turns. ICCs less than 0.4 were interpreted as poor, 0.4–0.8 as fair to good and more than 0.8 as excellent.

Results of (b) were used to depict fluctuations of gait parameters and evolution of CoVs during the trial by plotting cumulative values of 3 up to 40 (32) strides. Pearson’s correlation coefficients were calculated for individual cumulative means/CoVs over gait cycles 3–40 (32) with the individual mean/CoV at stride 40 (32). Respective R was plotted per stride.

Matlab 9.1 (Mathworks, Natick, MA, United States) with custom scripts was used for all further analyses.

## Results

### Clinical Features of Study Population

Parkinson’s disease patients were on average 60 ± 9 years old, had an average BMI of 25 ± 2 kg/m2, and scored 16 ± 4 (of 108) in the UPDRS-III. ET patients were 67 ± 10 years old, had a BMI of 25 ± 2 kg/m2 and scored 19 ± 10 (of 116) in the TRS score. Ataxia patients were 58 ± 7 years old, had a BMI of 25 ± 3 kg/m2 and scored 10 ± 5 (of 40) on SARA score. Detailed information about demographics and clinical parameters of respective cohorts are provided in Table 1.

### Detection of Turns

When considering all walks from disease groups, all 124 (=31 × 4; 100%) turns were detected, segmented and excluded from further analysis by the manufacturer’s algorithm (El-Gohary et al., 2013).

In HE, visual inspection revealed non-detection of turns in 10 of 172 individuals (5.8%, for an example see Figure 1A). Subjects in which non-detection of turns had occurred, as a group differed from the remaining 162 in shorter SL at 80.4 ± 6.3%stature (*p* = 0.0006) and longer ST at 1.088 s ± 0.1 (*p* = 0.01). More importantly, their CoVs were significantly higher compared to datasets with properly detected turns [CoV-SL of 6.5% ± 3.0 (*p* < 0.0001) and CoV-ST of 6.4% ± 5.0 (*p* < 0.0001)] which is likely due to confounding influence of steps in turn being integrated to calculation of results. Datasets including non-detected turns were excluded from further analysis.

### Gait Parameters Excluding Turns

In the remaining dataset (Table 2), stride length was expectedly shorter and stride variability higher in patients compared to HE. CoV-SL and CoV-ST were highest in the subgroup of ataxic subjects. Subgroup differences were not statistically evaluated as this was not the focus of this study.

**Table 2:** Stride length and time: mean, standard deviation, and coefficient of variation (CoV) in included cohorts.

Parameter	HE (*n* = 162)	MD_ALL_ (*n* = 31)	ATX subgroup (*n* = 7)	ET subgroup (*n* = 12)	PD subgroup (*n* = 12)
Stride length [% stature]	86.13 ± 4.83	76.99 ± 7.04	76.06 ± 7.55	78.50 ± 6.66	76.02 ± 7.45
CoV Stride length [%]	1.99 ± 0.90	3.41% ± 2.17	5.61% ± 3.30	2.74% ± 0.87	2.81% ± 1.47
Stride time [second]	1.02 ± 0.06	1.04 ± 0.11	1.12 ± 0.20	1.02 ± 0.07	0.99 ± 0.05
CoV Stride time [%]	2.10 ± 0.68	3.54% ± 2.22	5.65% ± 3.64	3.14% ± 1.13	2.70% ± 1.07

*CoV – Coefficient of variation; HE – healthy elderly; MD – neurological disorders with motor impairment, comprised of: ATX – patients with cerebellar ataxia; ET – essential tremor; PD – Parkinson’s disease.*

### Characteristics of Strides Before and After Turns

**FIGURE 2:**
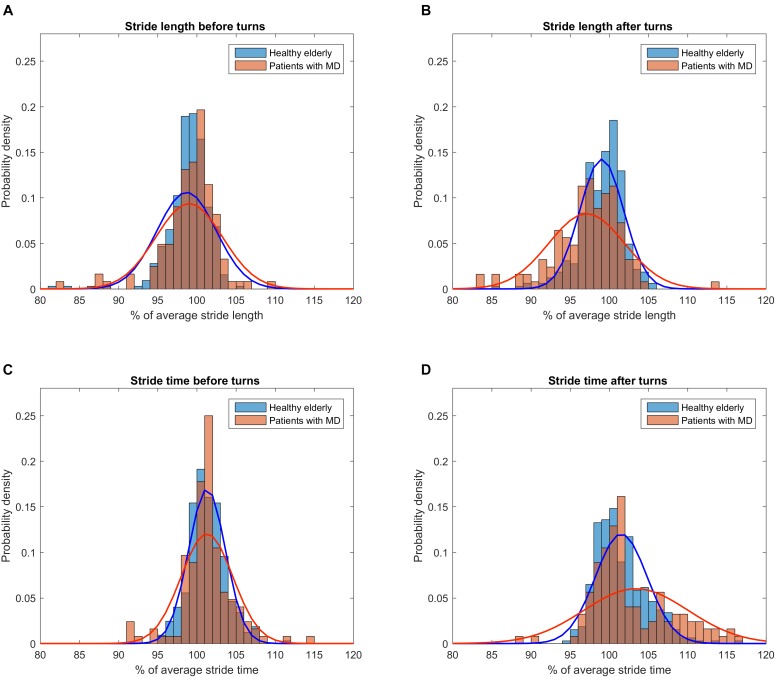
Distribution of gait parameter values right before and after turns relative to individual overall average. To account for different cohort sizes and number of turns, histograms were normalized for probability density. Parabolas depict the probability density function derived from means and standard deviations of the respective subset of strides. **(A)** Distribution of stride length values before turns for patients (red) and healthy subjects (blue). **(B)** Distribution of stride length values after turns. Note the wider spread of stride length values in the cohort of patients and the trend toward a shorter stride length compared to healthy subjects. **(C)** Distribution of stride time values before turns. **(D)** Distribution of stride time values after turns. Note the wider spread of stride time values in the cohort of patients and the trend toward a longer stride time compared to healthy subjects.

Lengths and times of strides before and after algorithmically excised turns were extracted and evaluated for systematic skew using a histogram/binning process (Figure 2). All patients were grouped into one MD group for clarity of depiction. This seems justified as we did not aim to explore differences between entities at this point but a technical issue in a group of subjects with suspected increase of CoV. In MD, 248 strides around turns were extracted (124 before and 124 after turns) and 648 strides were extracted from the HE dataset (324 before/after turns). Strides after turns were on average spatially shorter and temporally longer than means of 40 strides in MD patients (2.8 and 3.2% difference, *p* < 0.01) while in HE this applied to a lesser degree to strides before and after turns (1.2 and 1.4% difference, *p* < 0.0001, see Supplementary Figure S1). When described per group, alterations of stride characteristics were specifically prevalent in PD and ET groups for strides after turns (Supplementary Table S4).

### Gait Parameter Consistency Over Different Segments of 10/20 m of Straight Walking Between Turns

**FIGURE 3:**
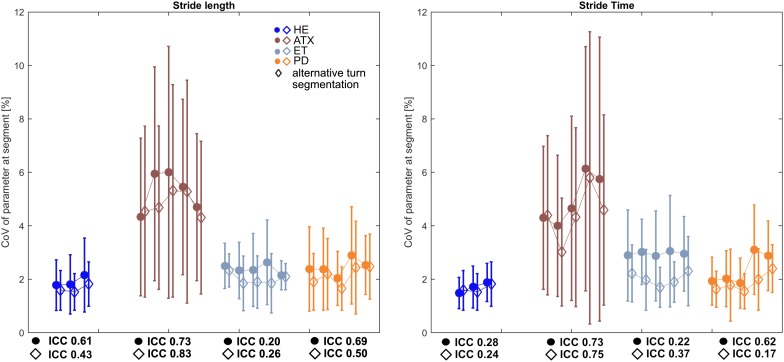
Characteristics of Intraclass Coefficients (ICC) for stride length and stride time CoVs calculated for each segment. Square markers depict averages of CoVs on group level for each segment, Whiskers indicate standard deviation (SD) of each segment. Diamond markers show averages of CoVs on group level after alternative turn segmentation of ±1 stride around turns. Respective ICC values for both approaches are stated underneath HE und patient subgroup graphs.

While stride length and stride time were highly consistent across the 3–5 segments (ICC > 0.90) in all groups (Supplementary Tables S1, S2), their CoVs showed only fair to poor consistency according to ICC (Figure 3). However, the range of absolute difference in CoVs across segments was smallest (less than 0.5%, see Supplementary Table S1 and Figure 3) in the HE and ET groups that also featured the lowest ICCs (Figure 3).

When this was recalculated after omission of one more stride before and after the manufacturer’s turn excision, this generally decreased CoV group means while respective ICCs only partially improved and range of CoV over segments only partially decreased (Figure 3 and Supplementary Table S2).

### Fluctuations of Gait Parameters Over the Gait Course

**FIGURE 4:**
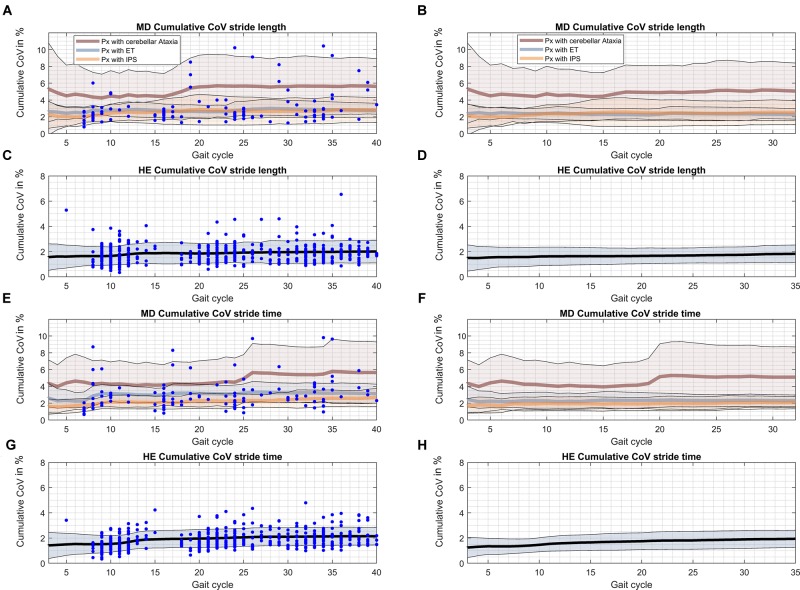
Cumulative CoVs for every gait cycle over the gait course. The occurrence of turns during the gait course is indicated by blue dots. Black – cumulative CoV of HE; brown – cumulative CoV of ATX; blue – cumulative CoV of ET; orange – cumulative CoV of PD. **(A)** Cumulative CoV stride length of patients with ataxia, essential tremor and Parkinson’s disease. **(B)** Cumulative CoV stride length of patients with ataxia, essential tremor and Parkinson’s disease after alternative turn segmentation. **(C)** Cumulative CoV stride length of healthy elderly. **(D)** Cumulative CoV stride length of healthy elderly after alternative turn segmentation. **(E)** Cumulative CoV stride time of patients with ataxia, essential tremor and Parkinson’s disease. **(F)** Cumulative CoV stride time of patients with ataxia, essential tremor and Parkinson’s disease after alternative turn segmentation. **(G)** Cumulative CoV stride time of healthy elderly. **(H)** Cumulative CoV stride time of healthy elderly after alternative turn segmentation.

The evolution of cumulative group means of CoVs along with respective confidence intervals over 40 gait cycles are depicted in Figure 4. While cumulative means of stride length and stride time were stable with constant confidence margins (Supplementary Figure S4) there were remarkable changes in CoV observed in single individuals of all patient groups. CoV analysis using manufacturer’s turn excision (Figures 4A,C,E,G) revealed sudden increases of individual cumulative means that were likely related to turning events. This resulted in a slight increase of CoVs over the course of gait cycles at group level and a widening of the confidence interval through to stride 40. However, absolute differences in CoVs after the 10th stride are rather of negligible magnitude at group level (up to 0.5% in patient groups, <0.2% in HE).

Using the explorative alternative turn segmentation (Figures 4B,D,F,G) and according total of 32 gait cycles seemed to attenuate but not totally eliminate this phenomenon. Specifically, confidence margins for CoV stride time still showed a sharp increase that could be narrowed down to performance of one ataxic subject at the individual level.

Pearson’s correlation coefficients of cumulative means/CoVs from stride 3 through to stride 40 versus the individual mean/CoV at stride 40 were plotted against strides. We used *R* > 0.8 as criterion to estimate the gait cycle N, at which each parameter can be reliably assessed (Table 3, Supplementary Figures S2, S3, and Supplementary Table S5). For stride length and stride time, very strong correlations (*R* > 0.9) were reached after only 3 gait cycles in all groups. For CoV stride length, *R* > 0.8 was reached at the 10th stride in ATX and PD group, but only after 20th stride in ET and 16th stride in HE. For CoV stride time criterion was reached at 8th stride for ATX, 18th and 19th stride for ET and PD and 20th stride in HE. These estimates were generally smaller for all patient groups when the alternative turn segmentation was applied (Supplementary Table S4, Supplementary Figures S2C,D, S3D–F). The correlation criterion indicated a number of < 10 strides as sufficient in the disease groups studied except for 16 strides for CoV stride length in ET. Estimates increased for HE (25 strides for CoV stride length and 17 for CoV stride time) possibly due to smaller total number of strides considered.

**Table 3:** Number of gait cycles needed reach correlation coefficient of *R* > 0.8 compared to 40 gait cycles in healthy elderly (HE) and subjects with movement disorders (MD).

	*N* to reach *R* > 0.8	Average at Nth GC (±SD)	Number (%) of subjects with increased CoV (>2.6%) at n	Average parameter after 40 GC (±SD)	Number (%) of subjects with increased CoV (>2.6%) at 40 GC
HE Stride length [%stature]	3	86.54 ± 5.01	n.a.	86.13 ± 4.83	n.a.
HE CoV Stride length	16	1.85% ± 0.86	21/162 (13%)	1.99 % ± 0.90	23/162 (14%)
HE Stride time [seconds]	3	1.029 ± 0.07	n.a.	1.021 ± 0.062	n.a.
HE CoV Stride time	20	1.90% ± 0.69	20/162 (12%)	2.10 % ± 0.68	30/162 (19%)
MD Stride length [%stature]	3	77.15 ± 6.94	n.a.	76.99 ± 7.04	n.a.
MD CoV Stride length	11	3.03% ± 1.69	17/31 (55%)	3.41 % ± 2.17	16/31 (52%)
MD Stride time [seconds]	3	1.02 ± 0.10	n.a.	1.032 ± 0.10	n.a.
MD CoV Stride time	10	3.02% ± 1.63	17/31 (55%)	3.54 % ± 2.22	19/31 (61%)
ATX Stride length[%stature]	3	76.18 ± 7.46	n.a.	76.06 ± 7.00	n.a.
ATX CoV Stride length	10	4.47% ± 2.79	5/7 (71%)	5.61 % ± 3.30	7/7 (100%)
ATX Stride time [seconds]	3	1.09 ± 0.14	n.a.	1.12 ± 0.20	n.a.
ATX CoV Stride time	8	4.28% ± 2.58	4/7 (57%)	5.65 % ± 3.64	6/7 (85%)
ET Stride length [%stature]	3	78.11 ± 6.73	n.a.	78.50 ± 6.66	n.a.
ET CoV Stride length	20	2.63% ± 0.72	5/12 (42%)	2.74 % ± 0.87	6/12 (50%)
ET Stride time [seconds]	3	1.021 ± 0.076	n.a.	1.02 ± 0.07	n.a.
ET CoV Stride time	18	3.20% ± 1.08	7/12 (58%)	3.14 % ± 1.13	7/12 (58%)
PD Stride length [%stature]	3	76.74 ± 6.41	n.a.	76.02 ± 7.45	n.a.
PD CoV Stride length	10	2.44% ± 0.97	4/12 (33%)	2.81 % ± 1.47	3/12 (25%)
PD Stride time [seconds]	3	0.979 ± 0.05	n.a.	0.99 ± 0.05	n.a.
PD CoV Stride time	19	2.24% ± 0.81	4/12 (33%)	2.70 % ± 1.07	6/12 (50%)

*ATX – patients with ataxia; CoV – coefficient of variation; ET – patients with essential tremor; GC – gait cycle; HE – healthy elderly; MD – neurological disorders with motor impairment; PD – patients with Parkinson’s disease; SD – standard deviation.*

## Discussion

Increased gait variability is a clinically relevant sign with potentially relevant implications for patient counseling in various neurological conditions and aging. As gait variability is not easily quantifiable from clinical observation, the instrumental assessment of gait is a valuable adjunct to clinical examination. However, the clinical use of such assessment is hampered by different constraints. Walking with 180° turns is a common paradigm to increase the number of recorded strides at the same time avoiding stops and establish a more steady-state-walking pattern. While widely applied wearable sensors in principle allow continuous gait monitoring, there remains uncertainty about the influence of assessment paradigms and environment on parameter output. For clinical use, the algorithms implemented for turn excision claim to separate straight, steady state walking from turning. We investigated a possible effect of using such turning paradigm with continuous kinematic recording on the assessment of gait variability from “regular” straight walking segments in groups of subjects who are known to have increased gait variability.

Our main findings can be summarized as follows: (1) algorithmic excision of turns can fail and result in misleadingly high values for gait variability and this seems to occur independent of gait dysfunction, (2) we observed indeed an effect of turns on spatiotemporal stride characteristics calculated of straight walking segments inbetween turns, which resulted in increased CoV and wider confidence margins with increasing number of steps, (3) despite low repeatability according to ICC, only marginal absolute changes in parameters of gait variability occur at group level between the 10th and 40th stride, indicating that the assessment of gait variability can be reliably performed using short distance walks that include less than 15 strides.

Our results help to determine the clinimetric properties of gait variability and may have relevant implications for the clinical use of instrumental gait analysis. When using systems with automated turn segmentation, the signal features used by manufacturers may differ and are usually not disclosed to the user. For the system used here, our data suggest that non-detection of turns was related to turning around a pylon whereas it did not occur in any of the “sharp” turns performed in the three patient groups. In other words, there may be a limitation of such algorithms to detect turns with broader diameter and subsequently lower horizontal accelerations. As non-detection can relevantly shift individual CoV into even pathological ranges (Figure 1C), it is important to exclude its occurrence before the interpretation of test results. The simple plotting of step time series used here seems an easily applicable quality check. As a pro argument for instrumental gait analysis with automated cutting of turns, non-detection of turns seemed unrelated to disease-specific changes in turning performance in elderly subjects with PD, ATX and ET.

As expected, we found lower stride length and higher CoV stride time and CoV stride length at group level in patients compared to HE. These differences, especially the increase of gait variability measures, were most pronounced in ataxic subjects which is in line with previous findings (Moon et al., 2016). Further, gait variability seen in HE was expectedly low at group level, but means + 1 SD of 2.89 and 2.78% are somewhat higher than previously reported (Konig et al., 2014a, 2016a), which may be related to the age of our healthy cohort. According to published cut-off of 2.6%, CoV stride time for 40 gait cycles was above cut-off in more than half in ATX, about half of ET patients and lowest rates in PD (Table 3) which is in line with previous findings (Moon et al., 2016). However, upto 19% (30/162) of HE featured increase of CoV in pathological range even when cases with non-detection of turns were excluded. One may speculate that this elderly cohort is likely to contain proportions of incipient neurodegenerative disorders or comorbidities or medications to explain this.

Using the manufacturers turn excision we found changes in spatiotemporal parameters in strides before and after turns that obviously affect CoV results of straight walking inbetween turns. The higher prevalence of such strides in a combined patient group of MD compared to HE group point to a disease-related phenomenon, specifically in PD and ET, though to be interpreted with caution due to small group sizes. It is conceivable that hesitation in step initiation in PD or postural adjustment after turn in ATX may result in spatially shorter and temporally longer strides after turns. If so, steps before or after turn contain disease-relevant information and optimizing turn segmentation algorithms might lead to loss of information. However, it is not precluded that the algorithmic turn detection gets imprecise due to disease-specific features like decreased turning velocity or trunk accelerations toward end of turn. Clarification of this point would warrant comparison against start and end of turn defined by a clinical observer which was not part of our protocol. In line with this, the exploratory approach of a more generous turn excision applied here, also remains arbitrary and excluding two steps rather than one before/after turns detected by manufacturer’s algorithm might also be discussed in the same right (see Figure 1C). Even if reliability could possibly be improved by a more generous turn excision, such adaptation will not usually be feasible in the context of clinical application.

Although absolute changes in CoV induced by the issue of turn segmentation are minor, this additional variance has implications for the definition of the repeatability of these parameters. The recommended > 50 steps for analysis of gait variability is based on the statistical assumption that increased numbers of observations (here: strides) will lower the variability (here: CoV stride length/time) of test results. However, our observation contrasts this assumption and unexpectedly showed increases of CoV group means and higher confidence limits with more strides. As suggested from plots of cumulative means, this phenomenon very likely reflects changes in stride characteristics before and after excised turns and questions the validity of our 40-stride results as appropriate reference for consistency analysis. In this sense, if CoV for short distances do not correlate with values for 40 strides (acquired in turning paradigm), this does not necessarily imply that 40 strides would be the better choice. In fact, the proportions of subjects with CoV in pathological range are quite comparable to using cumulative means of 10-20 strides (Table 3). Another common problem with ICC is its deflation with low to very low within-group variability (Weir, 2005). This has also been observed with other parameters of physiologically low variability like sway measures in static posturography of healthy subjects (Mancini et al., 2012). In line with this, despite only poor to fair repeatability according to ICC in our HE group, for example, absolute differences of CoVs were minimal (<0.5%). Data in previous reports seem to support this notion (Rebula et al., 2013; Konig et al., 2014a; Schmitz-Hubsch et al., 2016). Still, such error should be considered in interpretation of “borderline” CoV results. Importantly, as reported by others (Lord et al., 2011a; Rennie et al., 2018), presence of gait pathology does not seem to influence the reliability of stride variability measures: even lower numbers of only 10 strides (compared to 16/20 in HE) seem sufficient to achieve reliable estimates of gait variability in a population with high gait variability (ATX). With respect to continuous gait recording over longer walking distance, it has to be acknowledged that other factors apart from turns may affect reliability in clinical populations, e.g., decrease in attentional effort or fatigue, which could argue for preferring a shorter distance for most standardized recordings.

## Conclusion

Algorithmic detection of turns can fail and this seems due to turning instructions, rather than gait pathology. Moreover, the precision of algorithmic segmentation of straight walking segments interrupted by turns remains debatable, as spatiotemporal stride characteristics immediately before and after turns were found to differ from the averages generated from the full walking distances. Possible explanations include imprecise algorithmic segmentation, anticipatory slowing before turns, and disease-related hesitation after turns. As a general comment, collecting larger numbers of strides (including turns and automated turn segmentation) does not necessarily provide more robust estimates of gait variability compared to shorter distance level walks without turns. Our findings have relevant implications for the execution and interpretation of gait analysis in clinical settings.

## Ethics Statement

This study was carried out in accordance with the recommendations of the IRB of Charité – Universitätsmedizin Berlin and the ethics committee of the Medical Faculty of the University of Tübingen. The protocol was approved by the IRB of Charité – Universitätsmedizin Berlin and the ethics committee of the Medical Faculty of the University of Tübingen. All subjects gave written informed consent in accordance with the Declaration of Helsinki.

## Author Contributions

DK, WM, ME, and TS-H conceptualized the study, performed the statistical analyses, and drafted and revised the manuscript. A-CM, KO, SD, and SN contributed through data acquisition or project coordination and reviewing the manuscript. FP, DB, and AK contributed to project organization and execution and review of analyses and manuscript.

## Conflict of Interest Statement

TS-H has received honoraria as a speaker from Rölke Pharma. The remaining authors declare that the research was conducted in the absence of any commercial or financial relationships that could be construed as a potential conflict of interest.

## Supplementary Material

FIGURE S1 Characteristics of turn-related parameter values per group. **(A)** Stride length, **(B)** Stride time. Asterisks refer to significance in respective paired (within group) or unpaired *t*-test (between groups). ^∗^*p* < 0.05; ^∗∗^*p* < 0.001; ^∗∗∗^*p* < 0.0001.

FIGURE S2 Pearson’s correlations of individual cumulative parameter averages at gait cycle (GC) n with the overall average after 40 GC in **(A)** patients with movement disorders and **(B)** healthy elderly. After alternative turn segmentation, correlations were calculated over 32 GC. **(C)** Patients with movement disorders after alternative turn segmentation **(D)** healthy elderly after alternative turn segmentation. While stride length and stride time averages after just a few steps show already excellent and persistent correlation with average after 40 GC, CoVs exhibit more fluctuations. We used the threshold of *R* > 0.8 to estimate the minimum number of strides to reliably measure CoVs for each parameter in each cohort (see also Table 3 and Supplementary Table S5).

FIGURE S3 Pearson’s correlations of individual cumulative parameter averages at gait cycle (GC) n with the overall average after 40 GC in patient subgroups **(A)** patients with ataxia **(B)** patients with essential tremor **(C)** patients with Parkinson’s disease. After alternative turn segmentation, correlations were calculated over 32 GC. **(D)** Patients with ataxia **(E)** patients with essential tremor **(F)** patients with Parkinson’s disease. We used a threshold of *R* > 0.8 to estimate the minimum number of strides to reliably measure CoVs for each parameter in each cohort (see also Table 3 and Supplementary Table S3).

FIGURE S4 Cumulative gait parameter value for every gait cycle over the gait course. The occurrence of turns during the gait course is indicated by blue dots. Black – cumulative parameter of HE; brown – cumulative parameter of ATX; blue – cumulative parameter of ET; orange – cumulative parameter of PD. **(A)** Stride length over trial of patients with ataxia, essential tremor and Parkinson’s disease. **(B)** Stride length over trial of patients with ataxia, essential tremor and Parkinson’s disease after alternative turn segmentation. **(C)** Stride length over trial of healthy elderly. **(D)** Stride length over trial of healthy elderly after alternative turn segmentation. **(E)** Stride time over trial of patients with ataxia, essential tremor and Parkinson’s disease. **(F)** Stride time over trial of patients with ataxia, essential tremor and Parkinson’s disease after alternative turn segmentation. **(G)** Stride time over trial of healthy elderly. **(H)** Stride time over trial of healthy elderly after alternative turn segmentation.

TABLE S1 Consistency of gait parameters and their coefficients of variation (CoV) across straight walking segments.

TABLE S2 Consistency of gait parameters and their coefficients of variation (CoV) across straight walking segments per disease subgroups AFTER omission of one additional stride before and after turns.

TABLE S3 Characteristics of strides before and after turns. Average refers to the means of all 40 gait cycles.

TABLE S4 Characteristics of strides before and after turns per disease groups. Average refers to the means of all 40 gait cycles.

TABLE S5 Number of gait cycles needed to reach a correlation coefficient of *R* > 0.8 compared to 32 gait cycles in subjects with movement disorders (MD) and healthy elderly (HE) per disease subgroups AFTER omission of one additional stride before and after turns.
