# Supplementary figures and images for: Less Is More – Estimation of the Number of Strides Required to Assess Gait Variability in Spatially Confined Settings
Source: Front Aging Neurosci. 2019 Jan 21;10:435. doi: 10.3389/fnagi.2018.00435 (PMC6348278; doi:10.3389/fnagi.2018.00435)

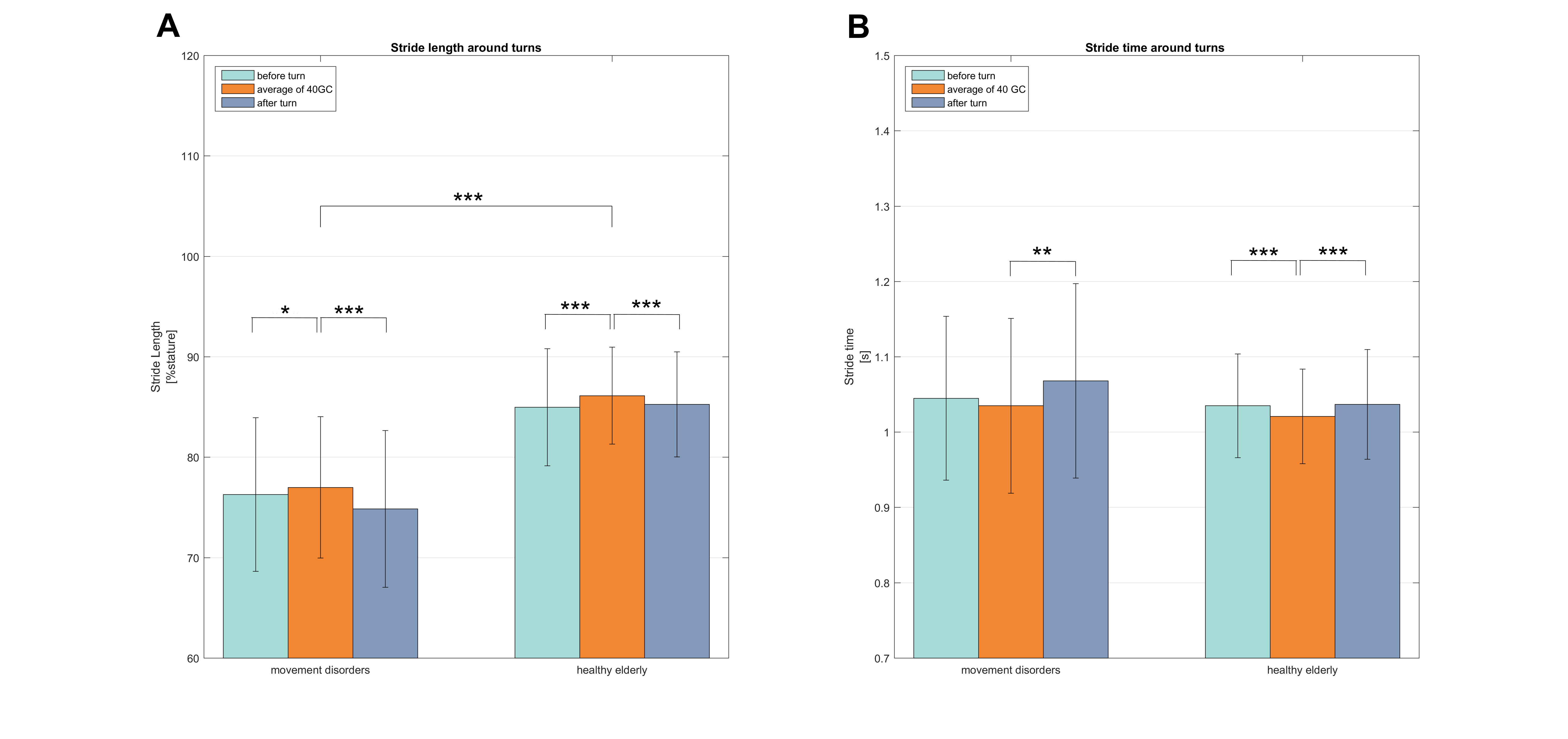

Supplement: FIGURE S1 — Characteristics of turn-related parameter values per group. (A) Stride length, (B) Stride time. Asterisks refer to significance in respective paired (within group) or unpaired t-test (between groups). ∗p < 0.05; ∗∗p < 0.001; ∗∗∗p < 0.0001. [file Image_1.TIFF]

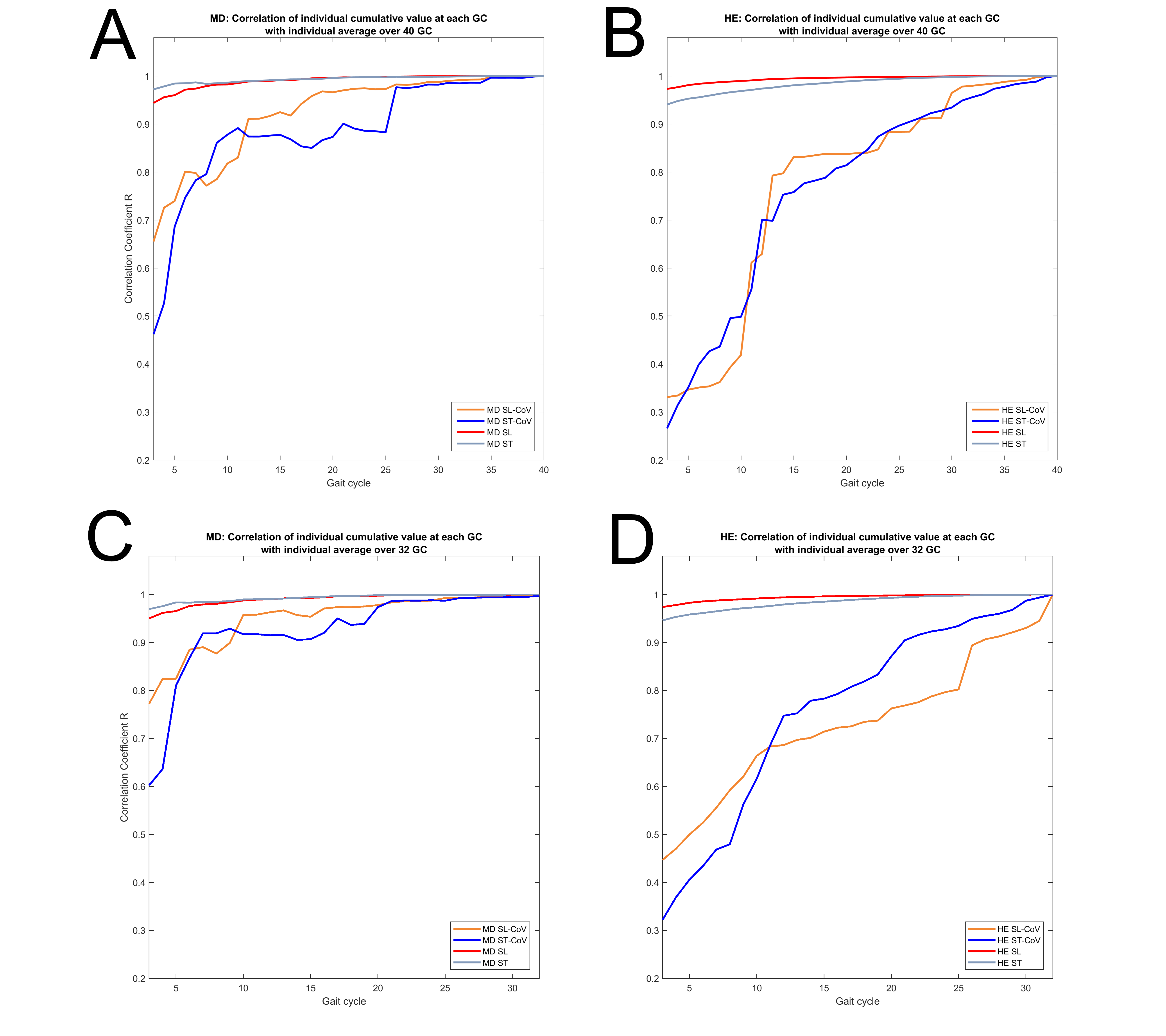

Supplement: FIGURE S2 — Pearson’s correlations of individual cumulative parameter averages at gait cycle (GC) n with the overall average after 40 GC in (A) patients with movement disorders and (B) healthy elderly. After alternative turn segmentation, correlations were calculated over 32 GC. (C) Patients with movement disorders after alternative turn segmentation (D) healthy elderly after alternative turn segmentation. While stride length and stride time averages after just a few steps show already excellent and persistent correlation with average after 40 GC, CoVs exhibit more fluctuations. We used the threshold of R > 0.8 to estimate the minimum number of strides to reliably measure CoVs for each parameter in each cohort (see also Table 3 and Supplementary Table S5). [file Image_2.TIFF]

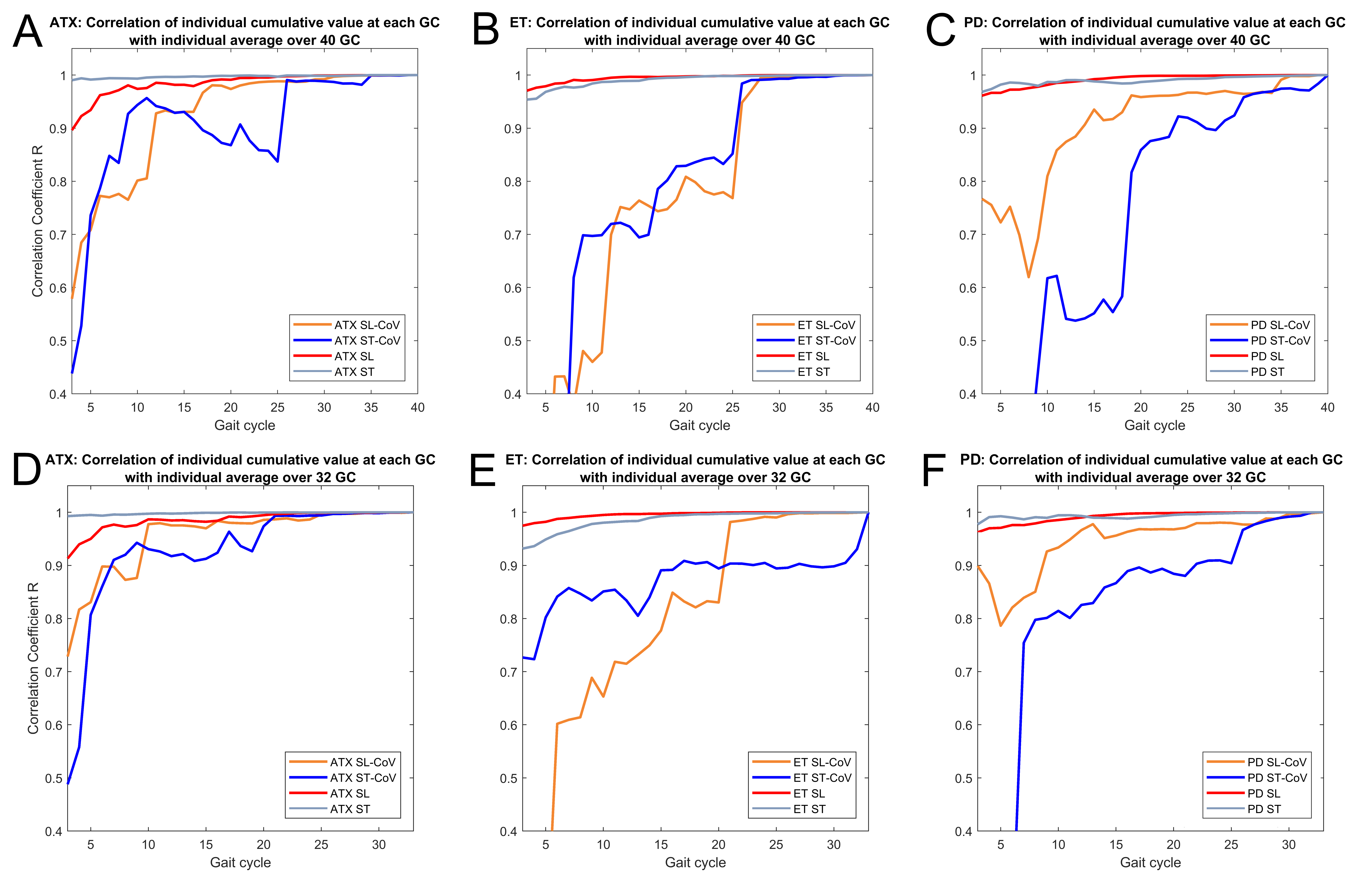

Supplement: FIGURE S3 — Pearson’s correlations of individual cumulative parameter averages at gait cycle (GC) n with the overall average after 40 GC in patient subgroups (A) patients with ataxia (B) patients with essential tremor (C) patients with Parkinson’s disease. After alternative turn segmentation, correlations were calculated over 32 GC. (D) Patients with ataxia (E) patients with essential tremor (F) patients with Parkinson’s disease. We used a threshold of R > 0.8 to estimate the minimum number of strides to reliably measure CoVs for each parameter in each cohort (see also Table 3 and Supplementary Table S3). [file Image_3.TIFF]

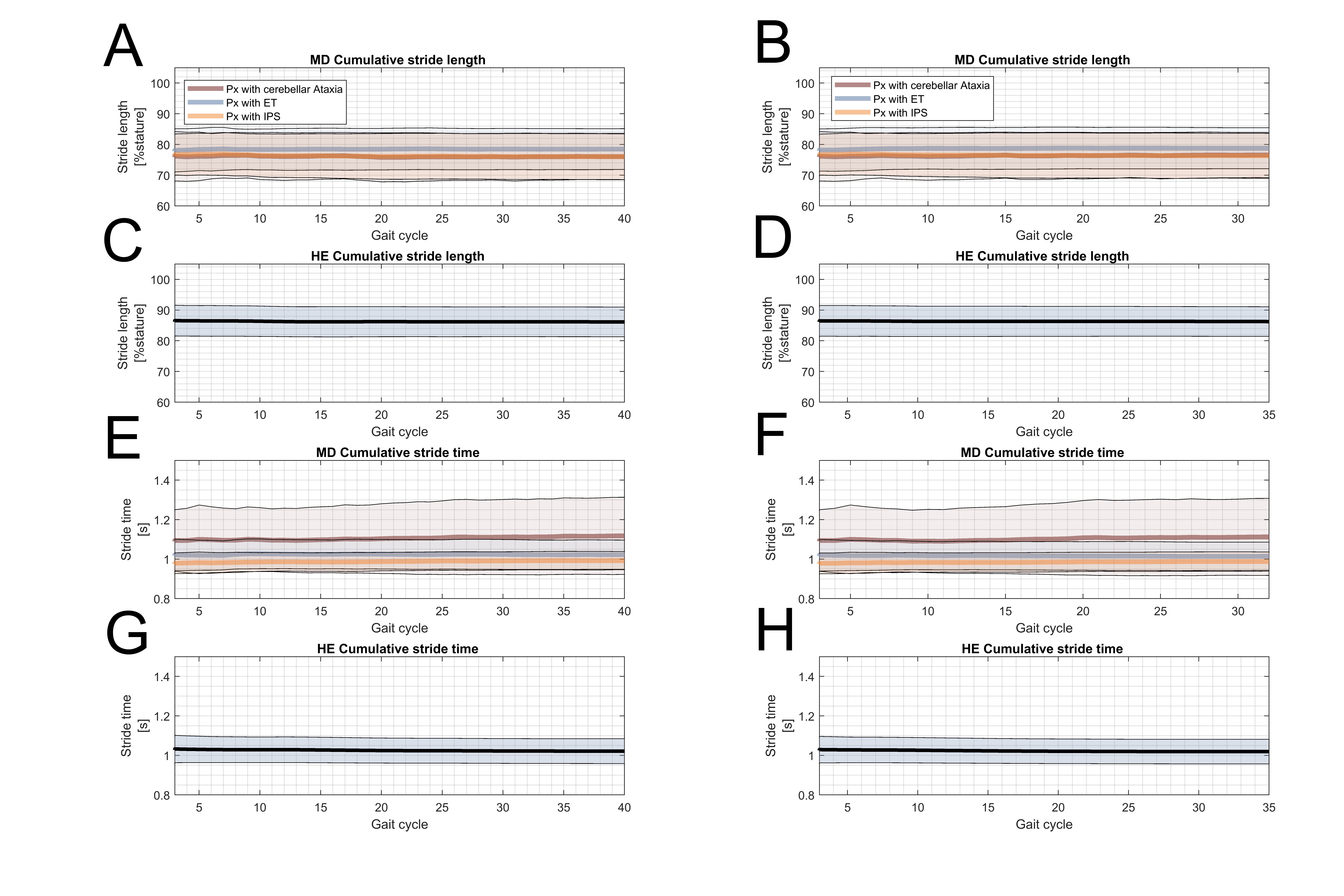

Supplement: FIGURE S4 — Cumulative gait parameter value for every gait cycle over the gait course. The occurrence of turns during the gait course is indicated by blue dots. Black – cumulative parameter of HE; brown – cumulative parameter of ATX; blue – cumulative parameter of ET; orange – cumulative parameter of PD. (A) Stride length over trial of patients with ataxia, essential tremor and Parkinson’s disease. (B) Stride length over trial of patients with ataxia, essential tremor and Parkinson’s disease after alternative turn segmentation. (C) Stride length over trial of healthy elderly. (D) Stride length over trial of healthy elderly after alternative turn segmentation. (E) Stride time over trial of patients with ataxia, essential tremor and Parkinson’s disease. (F) Stride time over trial of patients with ataxia, essential tremor and Parkinson’s disease after alternative turn segmentation. (G) Stride time over trial of healthy elderly. (H) Stride time over trial of healthy elderly after alternative turn segmentation. [file Image_4.TIFF]
