# Supplementary material for: Less Is More – Estimation of the Number of Strides Required to Assess Gait Variability in Spatially Confined Settings
Source: Front Aging Neurosci. 2019 Jan 21;10:435. doi: 10.3389/fnagi.2018.00435 (PMC6348278; doi:10.3389/fnagi.2018.00435)
Supplement: TABLE S1 — Consistency of gait parameters and their coefficients of variation (CoV) across straight walking segments. [file Table_1.pdf]

Supplementary Table 1 - *Consistency of gait parameters and their coefficients of variation (CoV) across straight walking segments*

**Healthy elderly**		**3 straight walking segments (each 20 meters)**		
	ICC	*p*	*Range of segment means (min - max)*	*SD of segment means*
**HE Stride length**	0.98	<0.0001	86.02 – 86.20	0.10
**HE CoV Stride length**	0.61	<0.0001	1.78 – 2.16	0.21
**HE Stride time**	0.94	<0.0001	1.02 - 1.03	0.01
**HE CoV Stride time**	0.28	<0.0001	1.48 – 1.90	0.16
**Movement disorders**		**5 straight walking segments (each 10 meters)**		
	ICC	*p*	*Range of segment means (min - max)*	*SD of segment means*
**MD Stride length**	0.97	<0.0001	76.76 – 77.37	0.46
**MD CoV Stride length**	0.69	<0.0001	2.86 - 3.37	0.75
**MD Stride time**	0.97	<0.0001	1.02 – 1.04	0.01
**MD CoV Stride time**	0.62	<0.0001	2.78 – 3.76	0.46
**ATX Stride length**	0.96	<0.0001	78.16 – 78.93	0.30
**ATX CoV Stride length**	0.73	<0.0001	4.33 - 6.01	0.75
**ATX Stride time**	0.98	<0.0001	1.10 – 1.14	0.02
**ATX CoV Stride time**	0.73	<0.0001	4.00 – 6.14	0.93
**ET Stride length**	0.98	<0.0001	75.64 – 76.69	0.43
**ET CoV Stride length**	0.20	0.028	2.15 – 2.63	0.18
**ET Stride time**	0.97	<0.0001	1.01 – 1.02	0.003
**ET CoV Stride time**	0.22	0.019	2.87 – 3.05	0.08
**PD Stride length**	0.97	<0.0001	76.76 – 77.37	0.25
**PD CoV Stride length**	0.69	<0.0001	2.86 – 3.37	0.21
**PD Stride time**	0.97	<0.0001	1.02 – 1.04	0.01
**PD CoV Stride time**	0.62	<0.0001	2.78 – 3.76	0.46

ATX – patients with ataxia, CoV – coefficient of variation; ET – patients with essential tremor; GC – gait cycle; HE – healthy elderly; ICC – intraclass coefficient; MD – neurological disorders with motor impairment; PD – patients with Parkinson's disease; SD – standard deviation

Supplementary Table 2 - *Consistency of gait parameters and their coefficients of variation (CoV) across straight walking segments per disease subgroups AFTER omission of one additional stride before and after turns*

**Healthy elderly**		**3 straight walking segments (each 20 meters)**		
	ICC	*p*	*Range of segment means (min - max)*	*SD of segment means*
**HE Stride length**	0.98	<0.0001	86.15 – 86.33	0.09
**HE CoV Stride length**	0.43	<0.0001	1.53 – 1.82	0.13
**HE Stride time**	0.94	<0.0001	1.016 - 1.027	0.006
**HE CoV Stride time**	0.24	<0.0001	1.37 – 1.62	0.11
**Movement disorders**		**5 straight walking segments (each 10 meters**		
	ICC	*p*	*Range of segment means (min - max)*	*SD of segment means*
**MD Stride length**	0.97	<0.0001	77.10 – 77.62	0.22
**MD CoV Stride length**	0.79	<0.0001	2.52 – 2.85	0.13
**MD Stride time**	0.97	<0.0001	1.020 – 1.033	0.006
**MD CoV Stride time**	0.70	<0.0001	2.14 – 2.86	0.33
**ATX Stride length**	0.95	<0.0001	76.26 – 77.11	0.33
**ATX CoV Stride length**	0.83	<0.0001	4.13 – 5.31	0.40
**ATX Stride time**	0.97	<0.0001	1.10 – 1.23	0.014
**ATX CoV Stride time**	0.75	<0.0001	3.02 – 5.79	0.88
**ET Stride length**	0.98	<0.0001	78.42 – 79.23	0.27
**ET CoV Stride length**	0.26	0.0067	1.85 – 2.14	0.12
**ET Stride time**	0.95	<0.0001	1.009-1.021	0.004
**ET CoV Stride time**	0.37	0.00053	1.70 – 2.30	0.20
**PD Stride length**	0.96	<0.0001	75.76 – 76.60	0.32
**PD CoV Stride length**	0.50	<0.0001	1.65 – 2.47	0.34
**PD Stride time**	0.93	<0.0001	0.98 – 0.99	0.004
**PD CoV Stride time**	0.17	0.051	1.49 – 2.40	0.33

ATX – patients with ataxia, CoV – coefficient of variation; ET – patients with essential tremor; GC – gait cycle; HE – healthy elderly; ICC – intraclass coefficient; MD – neurological disorders with motor impairment; PD – patients with Parkinson’s disease; SD – standard deviation

Supplementary Table 3 – *Characteristics of strides before and after turns. Average refers to the means of all 40 gait cycles.*

	**Mean**	**StD**	**p (vs. average)**	**% of stride values smaller than 97.5% of individual mean**	**Within 97.5%-102.5%**	**% of stride values greater than 102.5% of individual mean**
**HE SL BEFORE [%stature]**	84.98	5.825	< 0.0001	21.25	74.06	4.66
**HE SL AVERAGE [%stature]**	86.13	4.83	n.a.	n.a.	n.a.	n.a.
**HE SL AFTER [%stature]**	85.26	5.223	< 0.0001	24.07	69.14	6.79
**HE ST BEFORE [seconds]**	1.035	0.06897	< 0.0001	4.01	68.21	27.78
**HE ST AVERAGE [seconds]**	1.021	0.06276	n.a.	n.a.	n.a.	n.a.
**HE ST AFTER [seconds]**	1.037	0.07288	< 0.0001	5.56	61.42	33.02
**MD SL BEFORE [%stature]**	76.29	7.66	0.0182	22.31	69.42	9.02
**MD SL AVERAGE[%stature]**	76.99	7.04	n.a.	n.a.	n.a.	n.a.
**MD SL AFTER [%stature]**	74.85	7.81	<0.0001	47.58	45.53	7.26
**MD ST BEFORE [seconds]**	1.045	0.110	0.2829	6.45	68.55	25.0
**MD ST AVERAGE[seconds]**	1.035	0.116	n.a.	n.a.	n.a.	n.a.
**MD ST AFTER [seconds]**	1.068	0.130	0.0027	7.26	53.23	39.51

HE – healthy elderly; MD – neurological disorders with motor impairment; StD – standard deviation; ***n.a.*** *– not applicable, as comparisons of strides BEFORE and AFTER turns were made against averages of all strides.*

Supplementary Table 4 – *Characteristics of strides before and after turns per disease groups. Average refers to the means of all 40 gait cycles.*

	Mean	StD	p (vs. average)	% of stride values smaller than 97.5% of individual mean	Within 97.5%-102.5%	% of stride values greater than 102.5% of individual mean
**ATX SL BEFORE [%stature]**	74.01	9.19	0.1667	42.31	42.31	14.81
**ATX SL AVERAGE [%stature]**	76.06	7.56	n.a.	n.a.	n.a.	n.a.
**ATX SL AFTER [%stature]**	73.93	8.69	0.0514	50.00	37.04	14.29
**ATX ST BEFORE [seconds]**	1.12	0.19	0.5439	25.00	53.57	21.43
**ATX ST AVERAGE [seconds]**	1.12	0.20	n.a.	n.a.	n.a.	n.a.
**ATX ST AFTER [seconds]**	1.15	0.22	0.1697	17.86	50.00	32.14
**ET SL BEFORE [%stature]**	76.10	7.05	0.0021	21.43	75.00	3.57
**ET SL AVERAGE [%stature]**	78.50	6.66	n.a.	n.a.	n.a.	n.a.
**ET SL AFTER [%stature]**	78.27	7.02	0.4689	50.00	39.29	10.71
**ET ST BEFORE [seconds]**	1.07	0.11	0.0061	0	78.57	21.43
**ET ST AVERAGE [seconds]**	1.02	0.07	n.a.	n.a.	n.a.	n.a.
**ET ST AFTER [seconds]**	1.04	0.07	0.0001	0	35.71	64.29
**PD SL BEFORE [%stature]**	74.08	8.23	0.001	21.43	67.86	10.71
**PD SL AVERAGE [%stature]**	76.02	7.45	n.a.	n.a.	n.a.	n.a.
**PD SL AFTER [%stature]**	75.69	7.83	0.2364	42.86	50.00	7.14
**PD ST BEFORE [seconds]**	1.02	0.04	0.0224	0.00	64.29	35.71
**PD ST AVERAGE [seconds]**	0.99	0.05	n.a.	n.a.	n.a.	n.a.
**PD ST AFTER [seconds]**	1.01	0.05	0.0005	7.14	64.29	28.57

ATX – patients with ataxia; ET – patients with essential tremor; HE – healthy elderly; MD – neurological disorders with motor impairment; PD – patients with Parkinson's disease; SL – stride length; ST – stride time; StD – standard deviation; ***n.a.*** *– not applicable, as comparisons of strides BEFORE and AFTER turns were made against averages of all strides.*

Supplementary Table 5 –*Number of gait cycles needed to reach a correlation coefficient of R>0.8 compared to 32 gait cycles in subjects with movement disorders (MD) and healthy elderly (HE) per disease subgroups AFTER omission of one additional stride before and after turns.*

	**N to reach R>0.8**	**Average at nth GC (± SD)**	**Number (%) of subjects with increased CoV (>2.6%) at n**	**Average parameter after 32 GC (± SD)**	**Number (%) of subjects with increased CoV (>2.6%) at 32 GC**
**HE Stride length [%stature]**	3	86.48 ± 4.99	n.a.	86.25 ± 4.81	n.a.
**HE CoV Stride length**	25	1.69% ± 0.60	9/162 (5%)	1.80 % ± 0.67	17/162 (10%)
**HE Stride time [seconds]**	3	1.029 ± 0.07	n.a.	1.019 ± 0.062	n.a.
**HE CoV Stride time**	17	1.70% ±0.66	13/162 (8%)	1.91 % ± 0.70	22/162 (14%)
**MD Stride length [%stature]**	3	77.17 ± 6.77	n.a.	77.32 ± 6.84	n.a.
**MD CoV Stride length**	5	2.70% ± 2.04	10/31 (32%)	3.01 % ± 2.05	12/31 (39%)
**MD Stride time [seconds]**	3	1.02 ± 0.10	n.a.	1.026 ± 0.11	n.a.
**MD CoV Stride time**	6	2.56% ± 2.0	12/31 (38%)	2.87 % ± 2.06	10/31 (32%)
**ATA Stride length[%stature]**	3	76.18 ± 7.46	n.a.	76.60 ± 6.82	n.a.
**ATA CoV Stride length**	5	4.45% ± 3.39	5/7 (71%)	5.07 % ± 3.00	5/7 (71%)
**ATA Stride time [seconds]**	3	1.09 ± 0.14	n.a.	1.11 ± 0.18	n.a.
**ATA CoV Stride time**	5	4.44% ± 2.27	4/7 (57%)	5.08 % ± 3.27	4/7 (57%)
**ET Stride length [%stature]**	3	78.22 ± 6.58	n.a.	78.79 ± 6.37	n.a.
**ET CoV Stride length**	16	2.28% ± 0.64	4/12 (33%)	2.28 % ± 0.87	4/12 (33%)
**ET Stride time [seconds]**	3	1.019 ± 0.076	n.a.	1.01 ± 0.07	n.a.
**ET CoV Stride time**	5	2.24% ±1.12	6/12 (50%)	2.37 % ± 0.66	3/12 (25%)
**PD Stride length [%stature]**	3	76.69 ±6.38	n.a.	76.28 ± 7.03	n.a.
**PD CoV Stride length**	6	2.01% ± 1.04	2/12 (17%)	2.54 % ± 1.35	3/12 (25%)
**PD Stride time [seconds]**	3	0.98 ± 0.05	n.a.	0.99 ± 0.05	n.a.
**PD CoV Stride time**	9	1.92% ±0.77	2/12 (17%)	2.07 % ± 0.71	3/12 (25%)

ATX – patients with ataxia, CoV – coefficient of variation; ET – patients with essential tremor; GC – gait cycle; HE – healthy elderly; MD – neurological disorders with motor impairment; PD – patients with Parkinson's disease; SD – standard deviation
